# Effects of key parameters on pulsed field ablation of atrial fibrillation: potato experiments

**DOI:** 10.3389/fcvm.2025.1525762

**Published:** 2025-02-04

**Authors:** Yunhao Li, Fengqi Xuan, Daoyang Zhang, Mingyu Sun, Ping Zhang, Qi Zhang, Jie Zhang, Yaling Han, Zulu Wang, Ming Liang

**Affiliations:** ^1^ Department of Cardiology, General Hospital of Northern Theater Command, Shenyang, China; ^2^ Graduate School of China Medical University, China Medical University, Shenyang, China; ^3^ Department of Cardiology, Tianjin Chest Hospital, Tianjin, China; ^4^ Graduate School of Dalian Medical University, Dalian Medical University, Dalian, China; ^5^ National Key Laboratory of Frigid Zone Cardiovascular Diseases, Shenyang, China

**Keywords:** pulsed field ablation, key parameters, pre-clinical study, pulmonary vein isolation, tomato

## Abstract

**Aims:**

The study aims to investigate the impact of key parameters of pulsed field ablation (PFA) on lesion depth, including voltage (V), pulse width (PW), number of pulses (P), and ablation number (N), using potato models.

**Methods:**

Potatoes are utilized as a display of the irreversible electroporation lesion. The key ablation parameters were varied systematically to explore its influences on lesion depths.

**Results and conclusion:**

The key ablation parameters have varying degrees of influence on lesion depths, following the order of PW>V; V>P; PW>P; N>P. Lesion depths increased with higher values of V and P. However, when the total number of pulses reached 9,600, there was no significant increase in injury depth.

## 1 Background

Atrial fibrillation (AF) is the most common tachyarrhythmia requiring early clinical intervention (1). Catheter ablation is the most effective approach for treating AF (2), and conventional temperature-dependent energy sources, such as radiofrequency energy and cryo-energy, lack tissue selectivity, posing a risk of thermal damage to neighboring tissues. In contrast, pulsed field ablation (PFA) offers the advantage of non-thermal energy with tissue selectivity, with the ablation depth and width being determined by key parameter settings. However, the impact of each parameter range on ablation depth is not yet clear. In this study, we employed potatoes as a simulation model for the pulmonary vein to investigate the impact of key parameters of the pulsed field, such as voltage (V), pulse width (PW), number of pulses (P), and ablation number (N) on lesion depth. Through these evaluations, we aimed to provide valuable evidence for the preclinical study of PFA in the treatment of atrial arrhythmias.

## 2 Methods and materials

Potatoes that are selected from essentially uniform origin, variety, maturity, and dehydration level to ensure their electrical properties are consistent. They are then peeled and cut into pieces to reduce variability in thickness and to ensure a smooth surface. Pulsed field ablation system: Shanghai MicroPort EP MedTech Co., Ltd. Pulsed field ablation catheter (9-lead annular catheter): Shanghai MicroPort EP MedTech Co., Ltd.

**Figure 1:**
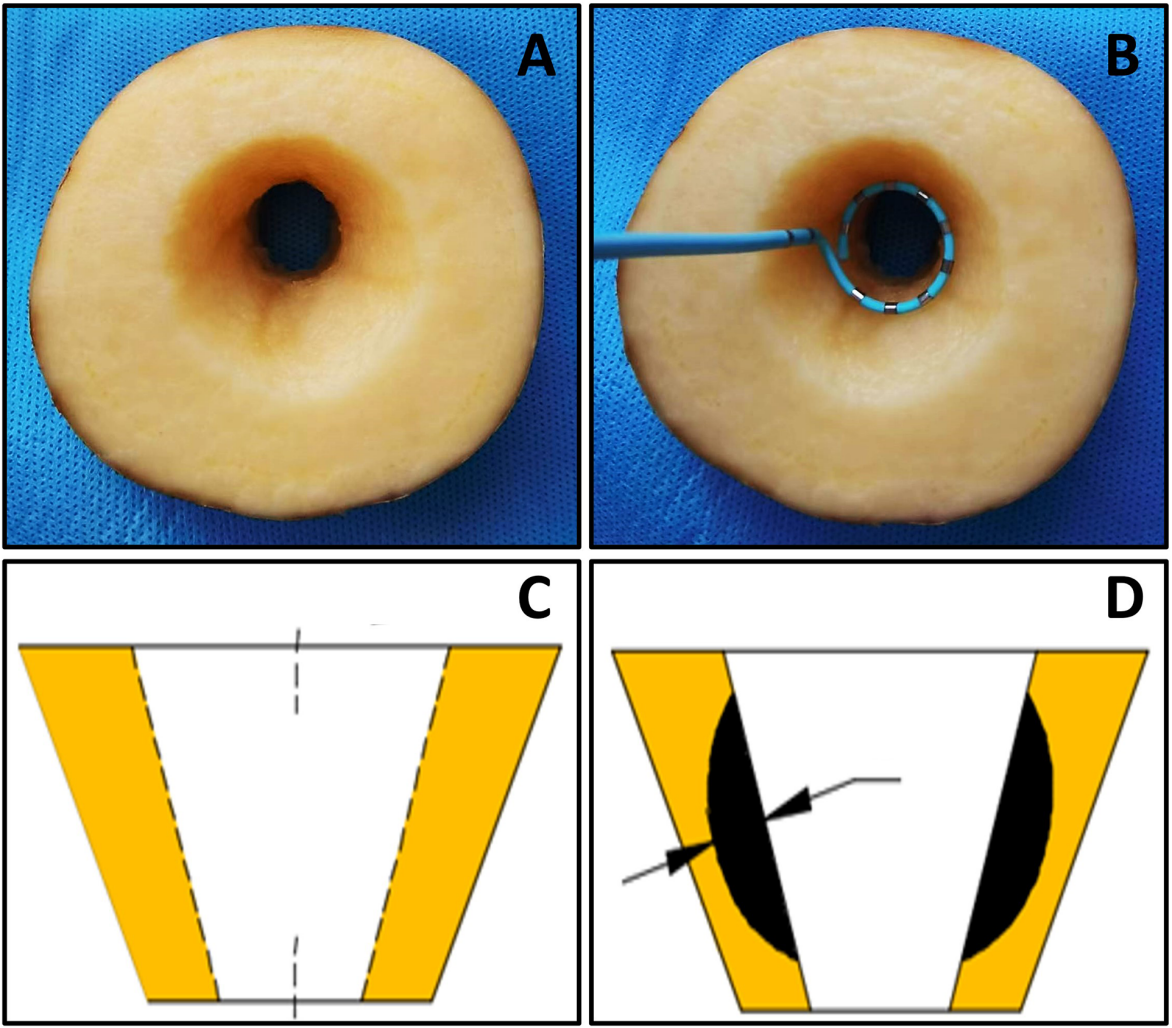
The potato model. **(A)** potatoes for ablation; **(B)** shows the position arrangement **(C,D)** measurement of ablation depth.

Fresh potatoes were cut into blocks of approximately 3 cm thickness, and a wedge-shaped groove structure with a maximum transverse diameter of approximately 4 cm was carefully excavated along the center of each tissue block. This groove structure was designed to simulate the anatomical characteristics of the pulmonary venous vestibule (Figure 1A) to assess the formation of irreversible electroporation (IRE) lesion depth (3, 4).

The preprocessed potato blocks are immersed in a 0.45% saline solution system to mimic blood impedance, with the temperature controlled at 37°C via a heating rod. A pump draws the liquid from the system and flushes it over the groove at a flow rate of 4 L/min, mimicking the blood flow in the pulmonary vein ostium. The ring catheter was placed vertically in the potato notch (the position arrangement is shown in Figure 1B) to investigate the effect of key parameters on lesion depth: The total pulse dose (V^2^ × PW × P × N) was constant, and V and PW were varied to set the 900V × 5 μs group and the 1,420 V × 2 μs group; V and P were varied to set the 1,000 V × 100 pieces group and the 1,300 V × 60 pieces group; PW and P were varied to set the 2 μs × 500 pieces group and the 5 μs × 200 times group; P and N were varied to set the 300 pieces × 12 times group and the 60 pieces × 60 times group. Furthermore, to investigate the impact of changes in each key parameters on ablation depths, we increased only the value of V while keeping other parameters fixed. We set 200 V group, 400 V group, 600 V group, 800 V group, 1,000 V group, 1,200 V group, 1,400 V group, and 1,600 V group. Only P × N was increased; we set 2,400 (number) group, 4,800 (number) group, 7,200 (number) group, 9,600 (number) group and 12,000 (number) groups. Each group was replicated at least 10 times. After ablation, the potatoes were stored in a wet box for approximately 24 h until the IRE area turned black and then the maximum ablation depths were measured with a caliper gauge (Figures 1C,D).

Mean ± standard deviation was used to express information that conformed to the normal distribution of measurement data, and median M (Q1, Q3) was used to express information that did not conform to the normal distribution. A paired samples *t*-test was used to compare the depth of injury indices at different time points. The one-way ANOVA analysis was used to compare the means between multiple groups, and the non-parametric test was used when the variance was not homogeneous. *P* value <0.05 was considered statistically significant. All statistical analyses were performed using SPSS 27.0 software.

## 3 Results

All the ablations were successfully conducted on potatoes. The mean ablation depths of the 900 V × 5 μs group and the 1,420 V × 2 μs group were 4.43 ± 0.16 mm and 4.11 ± 0.09 mm, respectively (*p* < 0.01), indicating that the PW had a greater influence on the ablation depths than the V (Table 1). The mean ablation depths of the 1,000 V × 100 pieces and 1,300 V × 60 pieces groups were 4.47 ± 0.27 mm and 4.88 ± 0.32 mm, respectively (*p* < 0.01), suggesting that the V had a greater influence than the P on the ablation depths (Table 1). The mean ablation depths in the 2 μs × 500 pieces and 5 μs × 200 pieces groups were 4.02 ± 0.18 mm and 4.28 ± 0.11 mm, respectively (*p* = 0.01), suggesting that the PW had a greater influence than the P on the ablation depths. The mean ablation depths in the 300 pieces × 12 times and 60 pieces × 60 times groups were 4.25 ± 0.19 mm and 5.06 ± 0.30 mm, respectively (*p* < 0.01), suggesting that the N had a greater influence than the P on the ablation depths. In summary, the degree of influence of each pulsed field parameter on ablation depth should be PW>V>P; N>P.

**Table 1:** The influence on ablation lesions under different parameters with the same total doses.

	V (V)	PW(μs)	P(pieces)	N(times)	Lesion depths(mm)	*P* value
V and PW	900	5	250	18	4.43 ± 0.16	<0.01
	1,420	2	250	18	4.11 ± 0.09	
V and P	1,000	5	100	36	4.47 ± 0.27	0.006
	1,300	5	60	36	4.88 ± 0.32	
PW and P	1,000	2	500	18	4.02 ± 0.18	0.01
	1,000	5	200	18	4.28 ± 0.11	
P and N	1,000	5	300	12	4.25 ± 0.19	<0.01
	1,000	5	60	60	5.06 ± 0.30	

**Figure 2:**
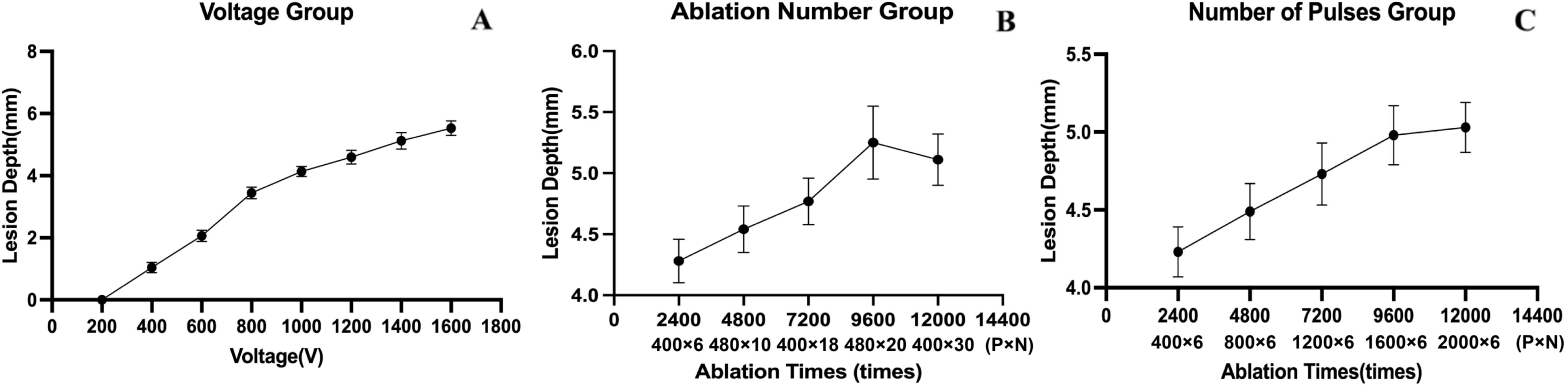
Depths varying with voltage, number of pulses and ablation number. The ablation depths did not significantly increase when the total number of pulses was more than 9,600, i.e., when the ablation depth reached the saturation point.

**Figure 3:**
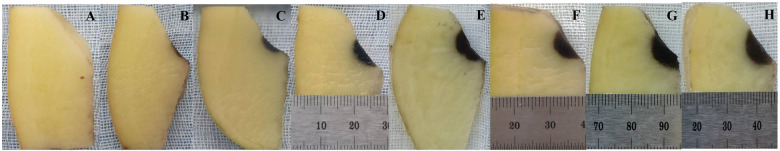
Different ablation depths of potatoes with different voltages. 200 V, 400 V, 600 V, 800 V, 1,000 V, 1,200 V, 1,400 V, 1,600 V for **A–H** respectively.

With other parameters kept constant and V was set to 200 V, 400 V, 600 V, 800 V, 1,000 V, 1,200 V, 1,400 V, 1,600 V, the corresponding ablation depths were 0 mm, 1.04 ± 0.17 mm, 2.06 ± 0.18 mm, 3.44 ± 0.19 mm, 4.14 ± 0.16 mm,4.60 ± 0.22 mm, 5.12 ± 0.27 mm, 5.53 ± 0.23 mm (*p* < 0.01) (Table 2, Figure 2). The ablation depths increased with increased V (Figure 3), while there was no obvious ablation injury when the voltage was <400 V. And when the voltage was increased to 1,600 V, obvious bubble was visible *in vitro*. With P controlled the same, by increasing the total numbers of pulses to 2,400 (400 × 6), 4,800 (480 × 10), 7,200 (400 × 18), 9,600 (480 × 20), 12,000 (400 × 30), the ablation depths were 4.28 ± 0.18 mm, 4.54 ± 0.19 mm, 4.77 ± 0.19 mm, 5.25 ± 0.30 mm, 5.11 ± 0.21 mm. With N controlled unchanged, by increasing the total number of pulses to 2,400 (400 × 6), 4,800 (800 × 6), 7,200 (1,200 × 6), 9,600 (1,600 × 6), 12,000 (2,000 × 6), the ablation depths were 4.23 ± 0.16 mm, 4.49 ± 0.18 mm, 4.73 ± 0.20 mm, 4.98 ± 0.19 mm and 5.03 ± 0.16 mm, respectively (Table 2, Figure 2). The ablation depths gradually increased as the total number of pulses increased, and both groups suggested that the ablation depths did not significantly increase when the total number of pulses was more than 9,600, i.e., when the ablation depth reached the saturation point.

**Table 2:** The influence on ablation depths with different voltages and different pulse number.

V(V)	PW (μs)	P (pieces)	N (times)	P × N (pieces)	Lesion depth (mm)	*P* value
200	2	The same	The same	9,600	0	<0.01
400	2			9,600	1.04 ± 0.17	
600	2			9,600	2.06 ± 0.18	
800	2			9,600	3.44 ± 0.19	
1,000	2			9,600	4.14 ± 0.16	
1,200	2			9,600	4.60 ± 0.22	
1,400	2			9,600	5.12 ± 0.27	
1,600	2			9,600	5.53 ± 0.23	
1,200	2	400	6	2,400	4.28 ± 0.18	<0.01
1,200	2	480	10	4,800	4.54 ± 0.19	
1,200	2	400	18	7,200	4.77 ± 0.19	
1,200	2	480	20	9,600	5.25 ± 0.30	
1,200	2	400	30	12,000	5.11 ± 0.21	
1,200	2	400	6	2,400	4.23 ± 0.16	<0.01
1,200	2	800	6	4,800	4.49 ± 0.18	
1,200	2	1,200	6	7,200	4.73 ± 0.20	
1,200	2	1,600	6	9,600	4.98 ± 0.19	
1,200	2	2,000	6	12,000	5.03 ± 0.16	

## 4 Conclusion

### 4.1 Assess PFA lesion by potatoes

Due to the difficulty in visualizing the ablation foci, the optimization of PFA parameters relies mainly on *in vivo* post-ablation experiments, which are challenging due to the complexity of *in vivo* settings and the large number of influencing factors (5). The approaches to measuring PFA lesion size for adjusting parameters are primarily categorized into *in vivo* and ex vivo methods. *in vivo* experiments, while capable of imitating human anatomical structures, are frequently disturbed by factors such as catheter contact force, cardiac pulsation, respiratory movement, and anatomical variations. Also, the operation procedures are more challenging (6, 7). The assessment of ablation efficacy or lesion sizes *in vivo* experiment is typically conducted through potential mapping, triphenyl tetrazolium chloride (TTC) staining, nitroso tetrazolium (NTZ) staining, and histological section measurement, each with inherent limitations. Post-ablation potential verification may be concealed by the “electrical shock” of the cell membrane, potentially resulting in a false “potential disappearance”. TTC and NTZ staining rely on the oxidative respiratory process of tissues for color displaying. They can be influenced by tissue hypoxia occurring during the time from animal euthanasia to tissue sampling (8, 9). Furthermore, the histological section is susceptible to distortion due to stretching, which could influence the reliability of measurements.

Ex vivo experiments allow the direct manipulation of catheters under visual control, ensuring consistent contact force with reduced equipment and technical expertise requirements. Nevertheless, the current evaluation methods are limited. Experiments on isolated myocardium, such as using ex vivo perfusion systems, are equipment and operationally demanding. Other methods, like using mouse cardiomyocyte cell lines or computer modelling to estimate cell mortality and predict lesion depths, have been explored, but the accuracy of these theoretical models requires further confirmation. Furthermore, TTC and NTZ staining, which depend on the oxidative respiratory process, are not suitable for evaluating ex vivo PFA ablation lesions. The potato model was initially used to adjust pulsed ablation parameters for tumor tissue. At 12 h post-ablation, the IRE zone in potatoes turned black due to oxidation, with a clear boundary with the surrounding normal tissue. This model is simple, controllable and suitable for repeated experiments. However, the response of potatoes to PFA may not be exactly equivalent to that of myocardium, and varietal differences may affect the response threshold. Nevertheless, studies have shown that the trends in the quantitative and qualitative effect relationships between ablation parameters and tissue injury are consistent between potatoes and myocardial tissue, allowing the use of a homogeneous potato model to approximate the extent of IRE injury (3).

### 4.2 The electric field strength and duration affect the depth of ablation

The electric field intensity and the duration of the pulsed field are the two most important factors determining the ablation depths of PFA. A higher field intensity induces more cell membrane electroporation, resulting in a higher transmembrane current and a more pronounced ablation effect. V, PW, electrode spacing and other factors can affect the field strength. In this study, the effects of different V on the injury depths were compared. When V reached 400 V, it could produce visible ablation injury (1 mm), while voltages below 400 V were ineffective. When reaching 1,000 V, the lesion depth reached 4 mm, which is sufficient for atrial ablation. When V increased to 1,600 V, the bubble formation and noise increased significantly. The voltage setting should be balanced between effectiveness and safety and adjusted according to different histological characteristics. The PW was found to have a greater effect on the injury depths than V. The results from the two groups, 2 μs × 500 pieces and 5 μs × 200 pieces, demonstrate that, given the same total PFA time (PW × P × N), extending the duration of a single application (increasing PW) leads to a more pronounced effect. An increased PW implies a longer duration for each PFA application. The study by Gintautas Saulis, et al. (10) suggests that the pore size of damage caused by microsecond pulses is smaller than that caused by millisecond pulses, which could be attributed to the pores having adequate time to expand under longer PW. Compared to PW = 2 μs, PW = 5 μs allows for a longer period after reaching the threshold for effect, resulting in greater material exchange across the cell membrane within each application, thus intensifying the damage. Consequently, the single PFA application duration (PW) may have a more significant impact on ablation depth than the electric field strength (V). Higher PW could induce stronger membrane perforation and better ablation effect, but the incidence of adverse reactions like myocardial fibrillation is also higher (11, 12). Current research suggests that microsecond PW optimizes the efficacy and safety of PFA. However, animal studies have shown that nanosecond pulsed waves reduced intraoperative ventricular arrhythmias, skeletal muscle and diaphragm tremors compared to the microsecond group (13). Since both PW and V affect membrane perforation in the same direction, an equivalent ablation effect can be achieved with a relatively low voltage when the PW is increased (12, 14).

Different combinations of V and PW have similar ablation effects but have certain safety differences. Therefore, these parameters should be combined organically according to the characteristics of the ablation site in order to balance effectiveness and safety. In addition to V and PW, the distance between the electrodes can also affect the field strength. The field strength decreases as the distance from the electrode increases. Importantly, the position between two electrodes has the weakest field strength, which makes it easy to form the ablation leakage point. As a result, the multi-electrode catheter is usually rotated appropriately at the original level after one discharge and then ablated again to cover the potential leakage between the electrodes.

The total pulse duration varies in the same direction as the ablation depth. In addition to the PW, the total pulse duration is mainly composed of the number of bursts and the delay time between bursts. The number of bursts is the product of the number of pulses delivered at one time and the number of discharges. Increasing the number of bursts increases the rate of membrane perforation and the depth of ablation (15). However, this study found that the relationship between the number of bursts and ablation depth is not purely linear. When the number of pulses is increased to a certain extent, the increase in ablation depth becomes less obvious, i.e., it reaches the saturated point. The saturation phenomenon observed in this PFA may be attributed to several factors: (1) The electric field intensity diminishes with increasing distance. Tissue at the periphery of the lesion may be subjected to an electric field that falls to the thresholds for reversible electroporation. While those tissues further away remain unaffected as they do not reach the electroporation threshold (16). Increasing the number of pulses extends the duration of the PFA without changing its intensity. Tissue within the reversible electroporation zone, benefiting from adequate exposure time and the cumulative effect of the electric field, remains in an electroporated state for a longer duration, leading to damage. Meanwhile, tissue that fails to reach the electroporation threshold remains largely unaffected, thus leading to a saturation effect (11). (2) After PFA application, the electric field attenuate more easily as it propagates through the damaged tissue (17). (3) Increasing the voltage can also result in marginal effects. When the voltage reaches a certain threshold, pop can be observed, suggesting that a portion of the electric field energy is lost as heat, leading to a reduction in the electric field energy (12).

The threshold for saturation with different parameter settings and its underlying mechanism has yet to be fully elucidated. Due to the presence of this saturation effect, there is a limit to increasing the number of pulse strings to enhance the ablation effect. Doing so may increase the risk of complications without significantly increasing the ablation depth. Interestingly, this study also found that the N had a more significant effect on ablation depth than the P, which suggests that increasing the number of discharges may achieve better clinical results when the total number of pulses is fixed.

Additionally, the delay between pulses may also have some effect on ablation. As the pulsed field passes through the tissue, it generates a certain amount of heat, which is usually negligible due to the effect of blood flushing, etc. However, if the delay between pulses is too short, the tissue may be “continuously charged”, generating a greater amount of thermal energy and increasing the risk of tissue temperature rise. Our study indicates that the magnitude of PFA energy is primarily influenced by the electric field intensity (V) and the duration (PW × P × N). The key parameters of PFA should be set through preclinical studies to fully balance their efficacy and safety. Our experiments show that when the voltage is increased to 1,600 V, a visible “pop” can be observed. Additionally, increasing the duration (PW, P, and N) will reach saturation at approximately 9,600 pulses. Therefore, exceeding this threshold by increasing the electric field intensity and duration may not significantly enhance the ablation effect while reducing safety.

The present study sought to evaluate the influence of the key parameters of PFA on the injury depths, and the relationships between the quantitative and qualitative effects. This was achieved through the use of the potato pulmonary vein vestibule model. Among the key parameters, PW had the greatest influence on ablation depth, followed by V and P. Interestingly, N had a greater influence on ablation depths than P. The ablation depth varied in the same direction as both V and the total number of pulses, but the injury depths saturated after the total number of pulses reached a certain value. The voltage and total number of pulses are the most frequently adjusted ablation parameters during clinical application. Understanding their effects on ablation depth is a guide to optimizing the adjustment of parameters in PFA. It is important to recognize that potato experiments can only offer limited information regarding the trends of how key parameters influence the depths of PFA lesions. The parameter settings derived from these experiments should not be directly applied to animal studies. Further optimization of these parameters will necessitate additional validation and adjustment through subsequent animal experiments and clinical trials. The findings of this study suggest that increasing voltage should must be with limits, and a saturation point exists for the total pulse number that can achieve maximum lesion depth. This insight provides valuable guidance for both animal experiments and clinical applications.

This study has a few important limitations that should be acknowledged. Firstly, the tissue types examined, namely potatoes, were somewhat different. As a result, the reflections of their respective trends on key parameters can only be used for estimation purposes and cannot be directly applied to myocardial tissue. Furthermore, as different pulsed systems have varying pulsed field parameter settings, catheter morphology, and electrode arrangements, some of the conclusions from this study may not necessarily be applicable to other pulsed ablation systems.

## Data Availability

The original contributions presented in the study are included in the article/Supplementary Material, further inquiries can be directed to the corresponding author.
